# Synthesis and Characterization of Activated Carbon Fibers Derived from Linear Low-Density Polyethylene Fibers Stabilized at a Low Temperature

**DOI:** 10.3390/polym13223918

**Published:** 2021-11-12

**Authors:** Kwan-Woo Kim, Hye-Min Lee, Seong-Hyun Kang, Byung-Joo Kim

**Affiliations:** 1 R&D Office 1st, Korea Carbon Industry Promotion Agency, Jeonju 54852, Korea; 01090063344@kcarbon.or.kr (K.-W.K.); leehm@kcarbon.or.kr (H.-M.L.); ksh8406@kcarbon.or.kr (S.-H.K.); 2 Department of Organic Materials & Fiber Engineering, Jeonbuk National University, Jeonju 54896, Korea; 3 Department of Carbon-Nanomaterials Engineering, Jeonju University, Jeonju 55069, Korea

**Keywords:** carbon fibers, recycling, upcycling, recovery, carbon fibers reinforced plastics

## Abstract

In this study, activated carbon fibers (ACFs) were prepared using a new method from polyethylene (PE) fibers. The stabilizing (or crosslinking) process of PE, an essential step, was achieved through a hybrid treatment using electron-beam/sulfuric acid at 110 °C that was more effective than the traditional method of using sulfuric acid at 180 °C for polyolefin. The stabilized precursor was then carbonized at 700 °C and activated at 900 °C with different activation times. The structural characteristics and morphologies of these ACFs were observed using an X-ray diffractometer and a field-emission scanning electron microscope, respectively. In addition, the N_2_/77K adsorption isotherm was used to discern textural properties. The total pore volume and specific surface area of these ACFs were found to be increased with a longer activation time, reaching final values of 0.99 cm^3^/g and 1750 m^2^/g, respectively. These ACFs also exhibited a high mesopore volume ratio (39%) according to crosslinking and crystallite formation conditions.

## 1. Introduction

Activated carbon fibers are generally manufactured using the same precursors as carbon fibers, such as synthetic polymers [1,2,3], petroleum-based pitch [1,4], and natural cellulose [1,5]. Specifically, activated carbon fibers are prepared through the stabilization, carbonization, and activation of precursor fibers. The activation can be achieved either by physical or chemical methods [6,7,8,9]. Physical methods use steam or carbon dioxide as an activation agent. They can be applied to most activated carbon fiber manufacturing processes because the process cost is low [10].

Activated carbon fibers have a high specific surface area and a well-developed pore structure. Therefore, they are widely used as environmental adsorption and energy storage materials [11,12,13]. However, since manufacturing activated carbon fibers requires many steps and high oxidation conditions, it usually entails a high cost. Thus, many studies have been conducted to find lower-cost manufacturing methods. The most common technique to produce activated carbon fibers is using new precursors [14,15,16].

Recently, carbon fiber has been produced using polyethylene, which is a relatively inexpensive material. Although carbon fibers produced with polyethylene have lower mechanical strengths than commercial carbon fibers, they show the possibility to manufacture carbon fibers using thermoplastic polyethylene [17]. To impart thermal stability to thermoplastic polyethylene, most studies have used sulfuric acid treatment at 180 °C or higher. However, since it is difficult to control the crosslinking rate at such high temperatures, precursor fibers are easily broken, making it challenging to develop a continuous process [18]. For that reason, polyethylene can be utilized as an optimal precursor for producing activated carbon fibers because thermal stability can be achieved using sulfuric acid at a lower temperature.

Typical well-known crosslinking methods for polyethylene include electron beam radiation [19], peroxide [20], silane coupling agents [21], and sulfuric acid treatment [22]. Sulfuric acid treatment can induce the highest crosslinking density, resulting in a high carbonization yield [18]. The crosslinking method using peroxide and silane coupling agents is unsuitable for crosslinking polyethylene fibers because it is performed on the surface with a limited depth of crosslinking [23]. However, irradiation of high-energy electrons can lead to sufficient crosslinking even with a fibrous precursor. In addition, electron beam treatment can easily implant radicals in the polyethylene chain structure [24]. When an electron beam and sulfuric acid treatment are applied together to polyethylene fibers, the electron beam can lead to the pre-curing of polyethylene chains. In addition, generated radicals can accelerate sulfuric acid crosslinking at a relatively low temperature [25].

In this study, we manufactured activated carbon fibers using a polyethylene precursor stabilized at a low temperature through electron beam irradiation and sulfuric acid complex treatment. We also determined the pore structure development in activated carbon fibers using different manufacturing conditions.

## 2. Experiment Details

### 2.1. Materials and Methods

**Figure 1:**
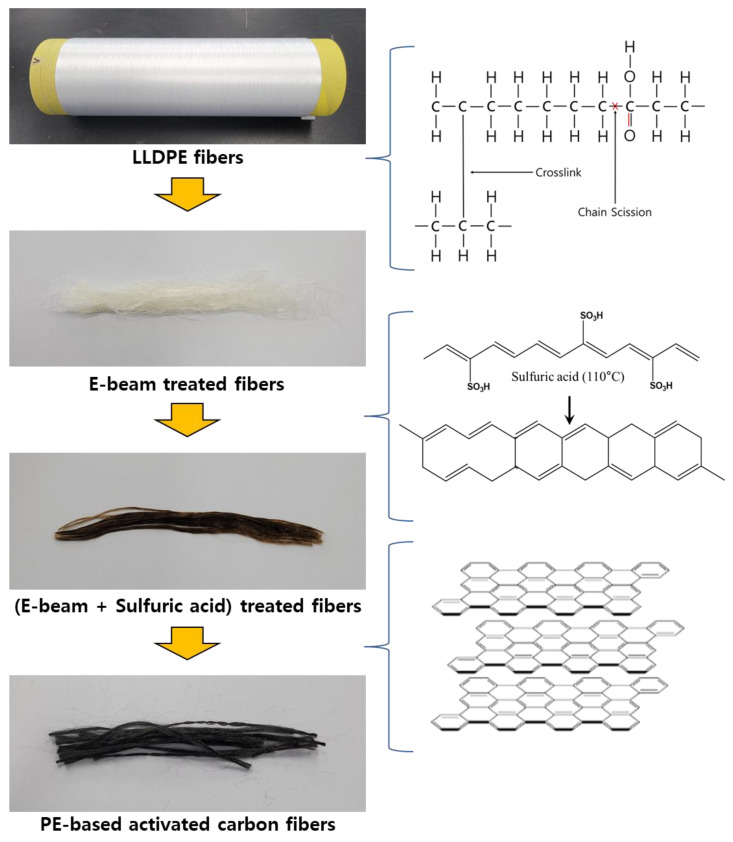
Manufacturing process of linear low-density polyethylene (LLDPE) based activated carbon fibers.

Linear low-density polyethylene (LLDPE, LG Chem., Seoul, Korea) and concentrated sulfuric acid (H_2_SO_4_, Daejung Chem., Siheung, Korea) were used in the experiment. First, an electron beam was stably irradiated on a flat stainless-steel plate (50 × 50 cm^2^). LLDPE fibers were placed on the plate, with both ends of the fiber fixed. Subsequently, the plate was irradiated using a 1.5 MeV accelerator (ELV-12, EB TECH Co. LTD., Daejeon, Korea) with a constant plate speed of 10 m/min. When passed once through the accelerator, the dose was ten kGy. The total dose was controlled at 1000 to 2000 kGy. The second crosslinking of LLDPE was carried out in hot sulfuric acid. A concentrated sulfuric acid (98%) was heated to a temperature of 110 °C. After treatments for a predetermined length of temperature and time, the LLDPE was taken out, washed thoroughly in distilled water, and dried in a drying oven at 60 °C for 24 h. The reaction mechanism of the hybrid crosslinking with LLDPE is illustrated in Figure 1.

These crosslinked LLDPE fibers were set on an alumina plate which was then inserted into a self-tuning alumina tubular furnace (length 1000 mm, SiC heater, diameter 100 mm, TENG, Jeonju, Korea). The crosslinked fibers were heated up to 900 °C at a temperature rise rate of 10 °C/min under a nitrogen gas flow and held at the carbonization temperature for one hour. The carbonization yield of the carbonized LLDPE fibers was found to be about 58.3%. The gas flow was then switched to H_2_O at a 0.5 mL/min rate and held for 20 to 40 min. The PE-ACF was then cooled under N_2_ gas at a flow rate of 300 mL/min [14].

The samples according to preparation conditions are listed in Table 1.

### 2.2. Characterizations

The effects of the crosslinking temperature on the characteristics of the samples were researched using thermal analyses such as thermogravimetric analysis (SHIMADZU, Kyoto, Japan) and differential scanning calorimetry (SHIMADZU, Kyoto, Japan). DSC analysis was performed at a heating and cooling rate of 10 °C/min with a temperature range of 30 to 300 °C. The sample was purged with nitrogen gas at a 10 mL/min flow rate to maintain an inert environment. TGA analysis was performed for all samples under a pure nitrogen atmosphere. All samples were heated from 30 °C up to 900 °C at a temperature rise rate of 10 °C/min.

Differences in the microstructure of the sample at each preparation step (E-beam treatment, carbonization, and activation steps) were determined using a X-ray diffractometer (PANalytical, Malvern, England) with an EMPYREAN X-ray diffractor having a customized auto-mount and a Cu K(alpha) radiation source at 30 mA and 40 kV. Diffraction patterns were investigated within diffraction angles from 10° to 90° at a speed of 2 °/min. Morphologies of hybrid-treated PE fibers and their ACFs were explored with scanning electron microscopy (SEM, AIS 2000C, Seron Tech Inc., Uiwang, Korea). To reduce charging during scanning electron microscopy imaging, samples were first placed on a sample holding plate and coated with platinum. When measuring, the pressure of the analyzer chamber was about 5 × 10^−5^ Pa, and the acceleration voltage was 20 kV.

The nitrogen adsorption isotherms of ACFs were measured with a BELSORP-Max (BEL Japan, Tokyo, Japan) at −196 °C (liquid nitrogen temperature). All samples were degassed for approximately 6 h at 301 °C, with the degassing pressure maintained at 0.1 Pa or less. The specific surface area was secured using the Brunauer–Emmett–Teller (BET) method [26]. Micropore and mesopore size distributions were estimated via the nonlocal density functional theory (NLDFT) [27] and the Barrett–Joyner–Halenda (BJH) [28] method, respectively.

A continuous flow column reactor (quartz column) was used to measure the acetaldehyde adsorption capacity of the PE-ACFs. Each ACF (0.5 g) was packed into the column (with a length of 700 mm and an inner diameter of 12.7 mm). The acetaldehyde (a flow rate of 2.0 L/min and a concentration of 10 μg/mL) was fed into the column. A gas detecting tube (92L, Gastech, Ayase-Shi, Japan) was used to monitor the concentrations of acetaldehyde at the outlet of the adsorption column.

## 3. Results and Discussion

### 3.1. Primary Crosslinking of Precursor Fibers

**Figure 2:**
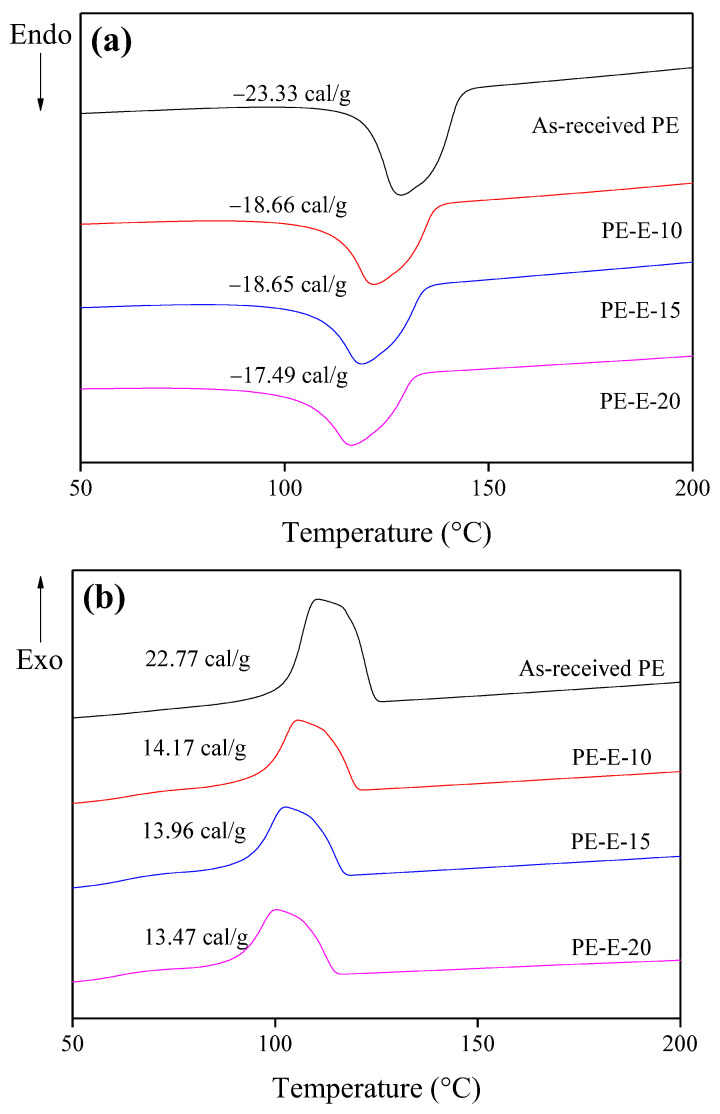
Differential scanning calorimetry (DSC) graphs of as-received LLDPE fibers and E-beam-treated fibers: (**a**) heating, (**b**) cooling.

The change in the calorific value of crosslinked LLDPE fibers according to the E-beam irradiation was measured by differential scanning calorimetry (DSC). The results are shown in Figure 2. It was confirmed that as the E-beam irradiation increased, the endothermic and exothermic values of the PE samples decreased. E-beam irradiation is known to induce crosslinking between PE chains and the rupture of C-C bonds. When the crosslinking between PE chains increases, the quantity of heat absorption required for melting and heat radiation by recrystallization will decrease. The results of this experiment were consistent with this theory.

In addition, it was confirmed that the maximum temperatures of T_m_ and T_c_ were reduced, indicating that samples were thermally unstable due to the rupture of C-C bonds in uncrosslinked chains caused by the E-beam irradiation as described above. Thus, melting and crystallization proceeded at a relatively lower temperature compared to the as-received PE.

**Figure 3:**
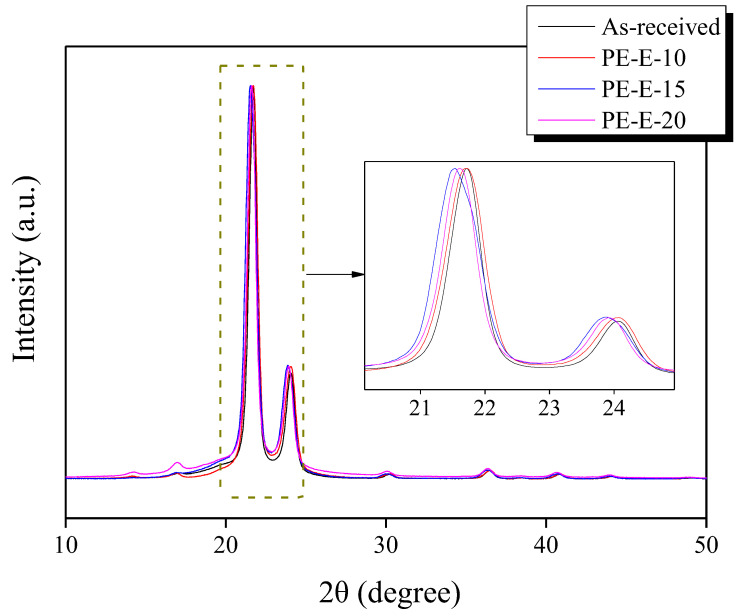
X-ray diffraction (XRD) patterns of as-received LLDPE fibers and E-beam crosslinked LLDPE fibers.

Differences in the microcrystalline structure of the PE fiber after different amounts of E-beam irradiation were observed through X-ray diffraction (XRD) analysis. The results are shown in Figure 3 and Table 2. As the E-beam irradiation increased to 1500 kGy, the crystallite size decreased. It rose again when the dose of irradiation was increased to 2000 kGy. It was presumed that the E-beam treatment induced crosslinking of the LLDPE molecular chain and caused the destruction of C-H and C-C bonds at the same time, resulting in a change of the crystallite size. When the irradiation dose was increased up to 1500 kGy, the average crystallite size was decreased in XRD because both crosslinking and the breaking of bonds occurred. When the irradiation dose was increased to 2000 kGy, which could be considered an excessive treatment, fine crystallites were mainly destroyed, and the average crystallite size was increased. These phenomena were considered to be related to decreases in T_m_ and T_c_ temperatures based on DSC results that occurred for the same reasons (i.e., crosslinking of the LLDPE molecular chain and destruction of C-H and C-C bonds).

### 3.2. Second Crosslinking in Sulfuric Acid

**Figure 4:**
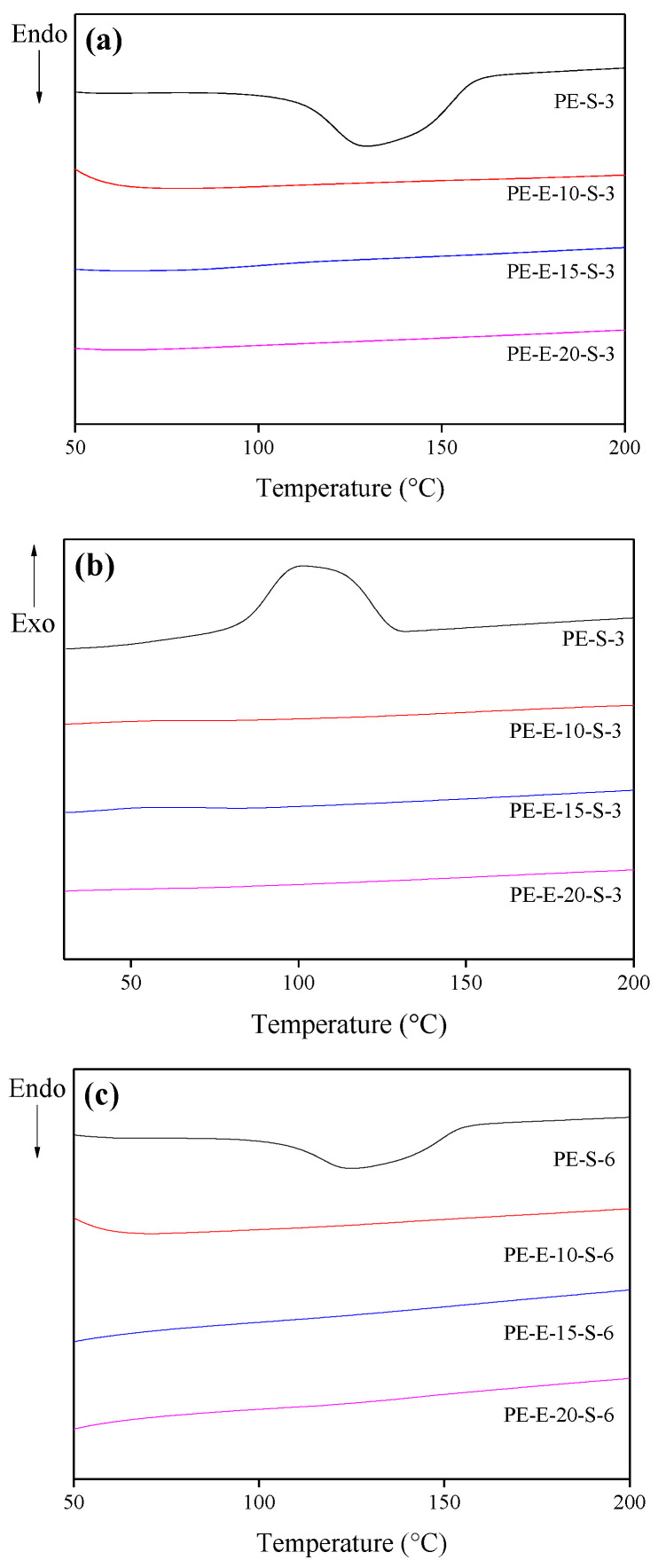

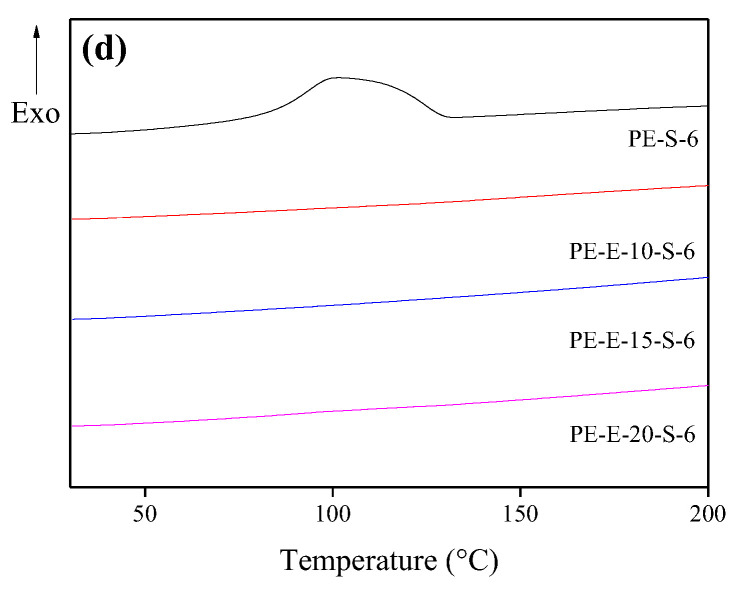
DSC graphs of E-beam- and sulfuric acid-treated LLDPE fibers: (**a**,**b**), sulfuric acid 30 min, (**c**,**d**), sulfuric acid 60 min.

**Figure 5:**
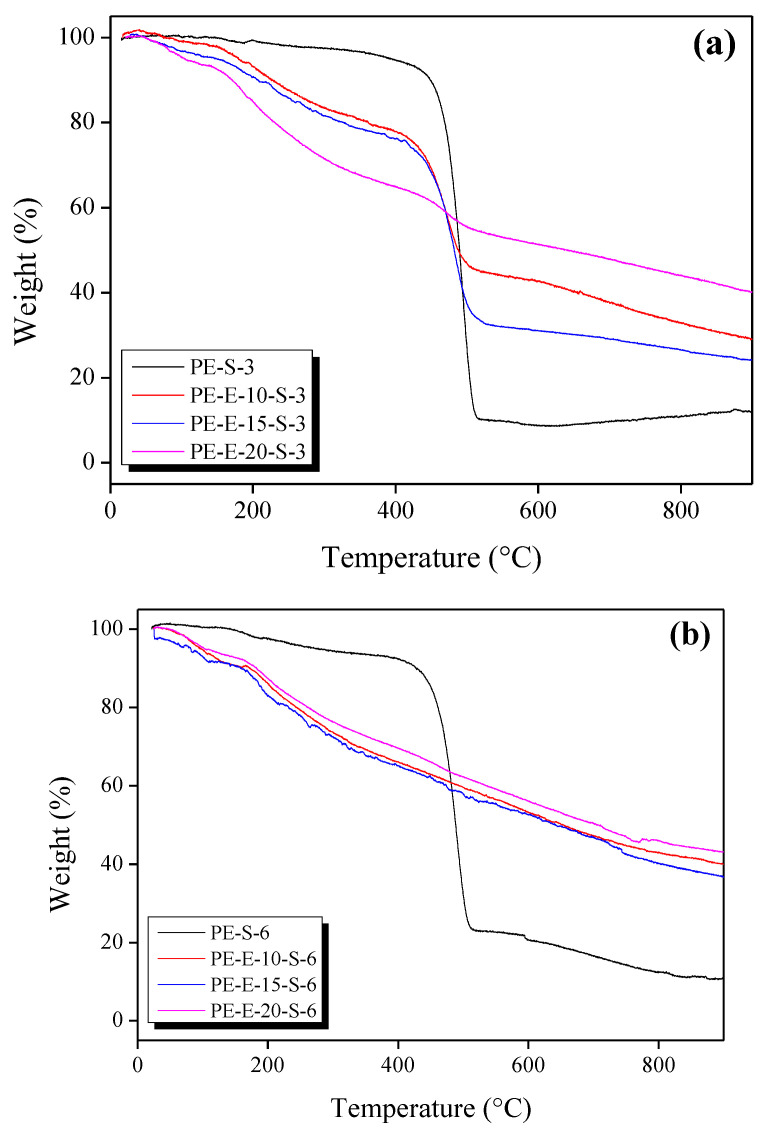
TGA graphs of E-beam and sulfuric acid treated LLDPE fibers: (**a**) sulfuric acid 30 min, (**b**) sulfuric acid 60 min.

As a second crosslinking step, pre-crosslinked LLDPE precursor fibers were treated in sulfuric acid for a different time period. DSC and TGA analyses were then performed for each sample after the second treatment. The results are shown in Figure 4 and Figure 5, respectively.

The secondary crosslinking was carried out by treating the first-treated precursor fibers in an undiluted sulfuric acid at 110 °C for 30 min or more. At this time, both ends of the fibers were fixed to minimize shape change (longitudinal direction) due to shrinkage. The DSC analysis showed that none of the secondary-treated samples exhibited endothermic or heat radiation behaviors required for melting and recrystallization. This meant that no molecular structure exhibiting thermoplastic behavior remained after the secondary treatment. Therefore, complete crosslinking or cyclization had occurred.

Figure 5 shows the results of the TGA analysis of the E-beam/Sulfuric acid hybrid-treated samples and the simple sulfuric acid-treated sample (comparative specimen). The most significant difference between the hybrid-treated samples and the comparative specimen was the thermal decomposition initiation temperature. The comparative sample exhibited a slight decrease in mass up to 450 °C, followed by a rapid decrease in mass, resulting in a final yield of about 10% (800 °C). On the other hand, all hybrid-treated samples steadily decreased in mass up to 800 °C (around 40% loss), reaching a final yield of about 45% (800 °C). The more significant initial decrease in mass exhibited by hybrid-treated samples as compared to the comparative sample was, therefore, determined to be due to the E-beam treatment. As noted previously, the E-beam treatment simultaneously induced crosslinking between PE molecular chains and ruptured C-C bonds, thus producing molecular structures with a low molecular weight and low thermal stability.

However, there was still a marked difference in weight between samples treated for 30 min and those treated for 60 min. In samples treated for 30 min, the initial mass loss was significant, and the final yield increased as the E-beam irradiation increased. This meant that dual effects of E-beam irradiation (molecular chain crosslinking and molecular chain cutting) occurred in proportion to its dose. All samples treated for 60 min exhibited similar TGA curves regardless of the E-beam irradiation dose. This meant that 60 min of sulfuric acid treatment was enough for complete crosslinking to occur.

### 3.3. Microstructure, Optic Image, and Morphology of Activated Carbon Fibers

**Figure 6:**
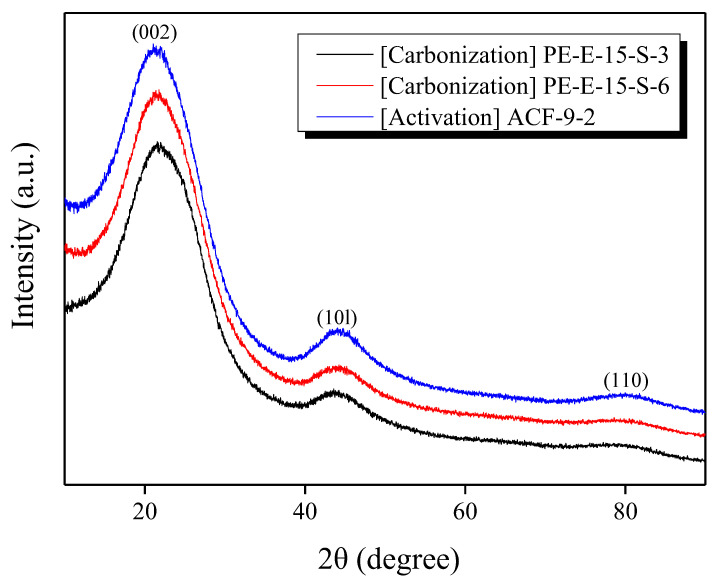
XRD patterns of hybrid-treated LLDPE fibers after carbonization (900 °C) and activation.

X-ray diffraction (XRD) is one of the methods that can efficiently analyze changes in the microcrystalline structure of carbon materials. Among hybrid-treated PE fibers, PE-E-15-S-30 and PE-E-15-S-60 samples were chosen to be carbonized at 900 °C in a nitrogen atmosphere. The carbonized PE-E-15-S-60 sample was then selected to be activated at 900 °C in a steam atmosphere for various time durations. The XRD patterns and calculated values for the two carbonized samples and the one activated sample (ACF-9-2) are depicted in Figure 6 and Table 3, respectively.

In the XRD pattern, typical 002 and 10l peaks of carbon materials were clearly observed for all samples. Both L_c_ (crystallite height) and L_a_ (crystallite size) increased significantly after carbonization and activation. This effect was due to the growth of crystallites themselves during the carbonization and activation process. It was also due to the fact that relatively small crystallites were preferentially oxidized. Thus, the average size was observed as increasing. Specifically, activation is a reaction of oxidizing graphitic crystallites of a carbonized precursor. It is widely known that oxidization occurs from amorphous domains of the precursor. In the activation process, carbonaceous precursors are oxidized in a particular order: first amorphous domains, then small crystallites, and then the edges of large crystallites. However, the L_a_ and L_c_ values calculated from XRD only conveyed the average values of all crystallites. Therefore, the increase in L_a_ was considered a relative increase due to the oxidation of amorphous domains or relatively small crystallites, and the increase in L_c_ was believed to be due to the oxidation of relatively small crystallites with few graphitic layers.

**Figure 7:**
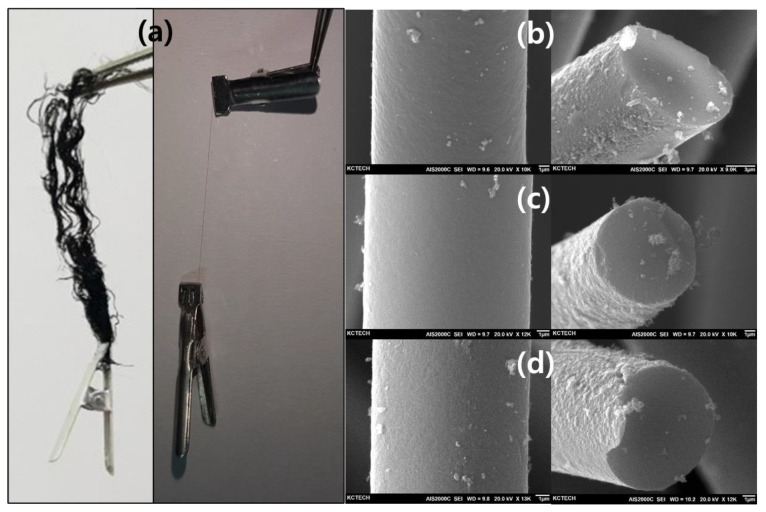
Optic and SEM images of activated carbon fibers: (**a**,**b**) ACF-9-2, (**c**) ACF-9-3, (**d**) ACF-9-4.

The optic and SEM images of the final activated carbon fibers (ACFs) are shown in Figure 7. The ACF-9-2 sample fiber maintained a hair-like shape. It was determined to have a strength of more than 13.6 MPa as it did not break even when 70 mg of the peg was attached to one ACF strand (average diameter of 8 um). The cross-section of each activated carbon fiber was observed in a circular shape through the SEM images. No damage was observed on the fiber surface. This was believed to be due to the very dense internal crosslinking through hybrid treatments.

### 3.4. Pore Characteristics of LLDPE-Based Activated Carbon Fibers

**Figure 8:**
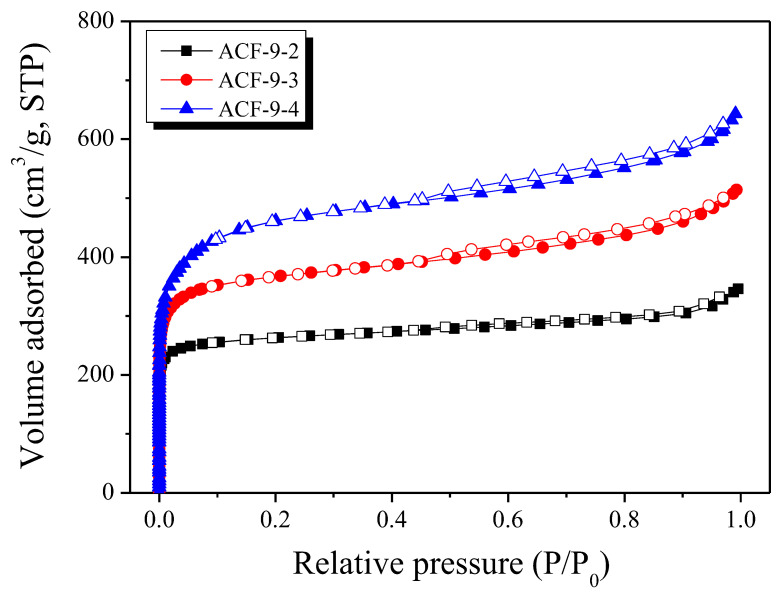
N_2_/77K adsorption/desorption isotherms of LLDPE-based activated carbon fibers as a function of activation time.

To analyze textural properties including the specific surface area, total pore volumes, and micropore volumes of the prepared PE-based ACFs, the isothermal adsorption/desorption curve was measured for each ACF sample. The results are shown in Figure 8. As the amount of nitrogen adsorption increased with an increasing activation time, the pore structure gradually became denser in proportion to the activation time. In addition, hysteresis became more pronounced with an increasing activation time, meaning that mesopores were formed at a slightly higher rate [29]. The isothermal adsorption/desorption curve of PE-based ACFs had the shape of Type-I based on the IUPAC classification. It was assumed that micropores were typically well-developed. However, near the relative pressure of about 0.05 P/P_0_, the slope of the isothermal adsorption/desorption curve decreased rapidly. It was determined that a significant portion of mesopores was included.

The textural properties of all ACF samples are summarized in Table 4. The specific surface area and total pore volume of PE-based ACFs were observed to be 1040~1750 m^2^/g and 0.53~0.99 cm^3^/g, respectively. The volumes of micropores and mesopores also increased as the activation time increased. It was confirmed that the mesopores’ volume (0.16~0.39 cm^3^/g) accounted for 30–39% of the total pore volume. This indicated that many mesopores appeared during the further activation process, which meant that many closed mesopores had already been generated by the oxidation of small crystallites during carbonization or activation. In addition, it was believed that the pore structure of ACFs could be controlled by controlling the crosslinking process. Such ACFs developed with controlled pore structures are expected to be suitable for use in various applications.

**Figure 9:**
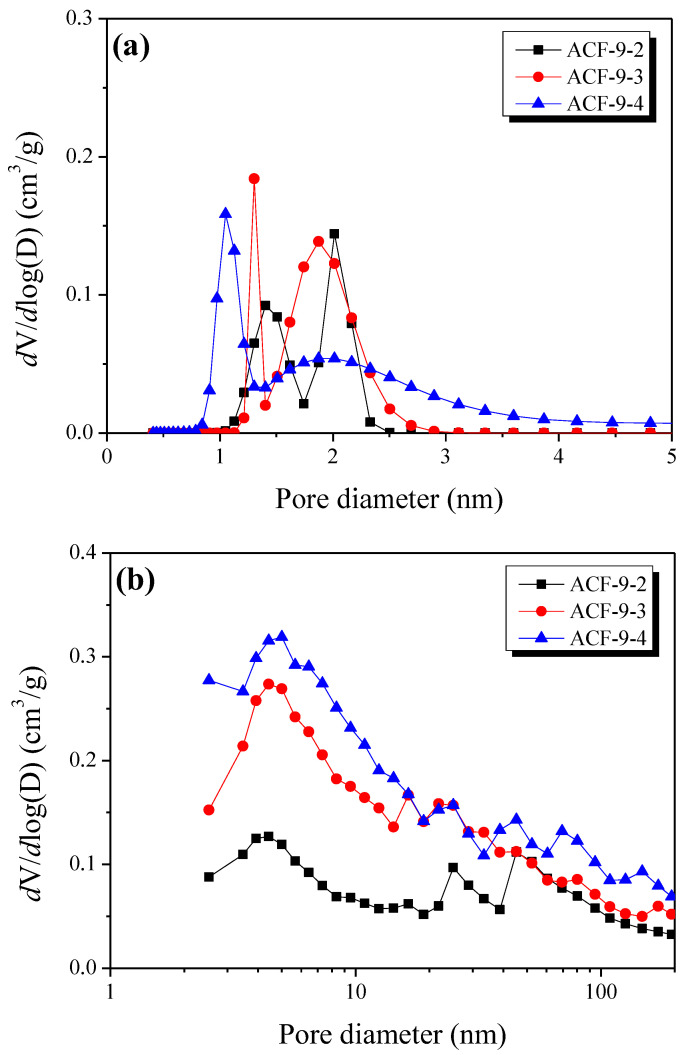
Pore size distribution curves of LLDPE-based activated carbon fibers as a function of activation time: (**a**) micropore, (**b**) mesopore.

The micropore and mesopore size distribution curves of PE-based ACFs were observed using NLDFT and BJH, respectively. The results are shown in Figure 9. The micropore distribution curves of PE-based ACFs revealed that ACF-9-2 mainly had over 1 nm of micropores and over 2 nm of mesopores. This is different from commercial ACFs, which generally have only well-developed micropores. In addition, it was observed that the distribution curve of micropores widened as the activation time increased. In the case of the mesopore distribution curve, it was confirmed that mesopores were well-developed within a wide range from 2 to 100 nm in all PE-based ACFs. It was also confirmed that the pore volume with such a range steadily increased with an increasing activation time. The above results concluded that PE-based ACF had both micropores (from the oxidation of amorphous and crystallite edges) and mesopores (from the oxidation of small crystallites resulting from the crosslinking process).

The adsorption behaviors of the ACF for harmful gases were observed by measuring the acetaldehyde breakthrough curves. Figure 8 exhibits the breakthrough curves of PE-based ACF and commercial ACF (FM10 was selected as a comparative sample, a cellulose-based activated carbon fiber from Chemviron from the UK. FM10 has a 1180 m^2^/g specific surface area, 0.5 cm^3^/g total pore volume, and 0.44 cm^3^/g micropore volume).

**Figure 10:**
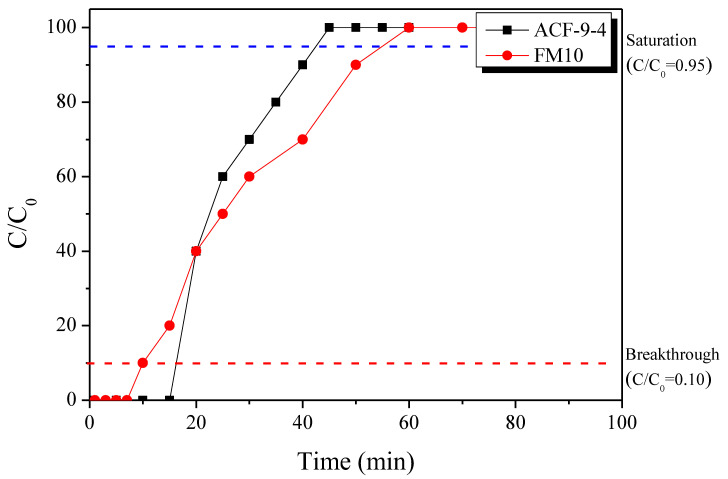
Breakthrough curves for the acetaldehyde adsorption of LLDPE-based activated carbon fibers.

PE-based ACF exhibited a longer breakthrough time than FM10 but a shorter saturation time (Table 5 and Figure 10). The acetaldehyde adsorption capacity of ACF-9-4 was 1.27 mg/g, which was observed to be about 164% of the acetaldehyde adsorption capacity of FM10. On the other hand, FM10 has more breakthrough capacity because the slope of the breakthrough curve is lower than that of ACF-9-4. In many previous studies, the acetaldehyde adsorption capacity was found to be highly correlated with the micropore volume of the adsorbent [30]. The values obtained by dividing the adsorption capacity by each micropore volume were confirmed to be 2.12 and 1.75, respectively. This means that the micropore volume is not the only factor determining the acetaldehyde adsorption capacity of the ACFs, but that the nature of the precursor or manufacturing can also affect their adsorption ability. Therefore, ACF-9-4 is considered to have a higher acetaldehyde adsorption performance than FM10 because it has an advanced porous structure and material characteristics.

## 4. Conclusions

In this study, linear low-density polyethylene (LLDPE)-derived activated carbon fibers (PE-ACF) were prepared by techniques of crosslinking, carbonization, and subsequent steam activation under various conditions. The LLDPE as a precursor was crosslinked by a hybrid process to obtain a high degree of carbonization. The total pore volume and specific surface area of the activated samples increased with a longer activation time to final values of 0.99 cm^3^/g and 1750 m^2^/g, respectively. The PE-ACF also exhibited a high mesopore volume ratio of 39%. The structural characteristics of the precursor LLDPE led to the production of ACFs with a mesopore-rich pore structure. The prepared PE-ACFs were confirmed to have better pore characteristics and aldehyde adsorption properties than the conventional commercial ACF. These results indicate that LLDPE can be a potential material for preparing activated carbon fiber precursors and that hybrid crosslinking shows potential for the development of PE-based carbon fibers.

## Figures and Tables

**Table 1:** Sample names according to crosslinking and activation conditions.

Sample Name	Crosslinking Conditions				Sample Name	Activation Conditions	
	E-Beam		Sulfuric Acid			Temp.	Time
PE-E-10-S-3 PE-E-10-S-6	1.5 MeV	1000 kGy	110 °C	30, 60 min	-	-	-
PE-E-15-S-3 PE-E-15-S-6		1500 kGy			ACF-9-2 * ACF-9-3 * ACF-9-4 *	900 °C	20 to 40 min
PE-E-20-S-3 PE-E-20-S-6		2000 kGy			-	-	-

* This sample was prepared using the PE-E-15-S-6 sample.

**Table 2:** XRD result of as-received LLDPE fibers and E-beam-treated fibers.

Sample Name	110 Peak				200 Peak			
	2θ	FWHM (2θ)	d_110_ (Å)	L_110_ (Å)	2θ	FWHM (2θ)	d_200_ (Å)	L_200_ (Å)
As-received	21.70	0.59	4.09	136.46	24.03	0.70	3.70	116.12
PE-E-10	21.69	0.71	4.09	114.06	24.02	0.81	3.70	99.94
PE-E-15	21.57	0.78	4.12	103.74	23.88	0.88	3.72	92.13
PE-E-20	21.60	0.65	4.11	125.16	23.90	0.76	3.72	107.54

**Table 3:** XRD patterns of hybrid crosslinked LLDPE fibers after carbonization and activation.

Step	Sample Name	002 Peak			10l Peak		
		2θ	d_002_ (Å)	L_c_ (Å)	2θ	d_10l_ (Å)	L_a_ (Å)
Carbonization	PE-E-15-S-3	22.46	3.96	9.04	44.33	2.04	17.76
	PE-E-15-S-6	22.34	3.98	9.25	44.52	2.03	17.48
Activation	ACF-9-2	22.50	3.95	9.70	44.80	2.02	30.40

**Table 4:** Pore characteristics of activated carbon fibers based on LLDPE manufactured with different activation times.

Sample Name	S_BET_ (m^2^/g)	V_Total_ (cm^3^/g)	V_Micro_ (cm^3^/g)	V_Meso_ (cm^3^/g)	Yield (%)
ACF-9-2	1040	0.53	0.37	0.16	55.2
ACF-9-3	1420	0.79	0.49	0.30	36.4
ACF-9-4	1750	0.99	0.60	0.39	10.0

**Table 5:** Breakthrough and saturation adsorption capacity of the LLDPE-based activated carbon fibers.

Gas	Breakthrough			Saturation	
	Time (min)	Adsorption Capacity (mg/g)	Adsorption Capacity/Micropore Volume (mg/cm^3^)	Time (min)	Adsorption Capacity (mg/g)
ACF-9-4	16.2	1.27	2.12	42.5	1.98
FM10	10.0	0.77	1.75	55.1	2.20

## Data Availability

Not applicable.
